# Integrated prediction of delayed graft function after kidney transplantation using the renal artery resistive index and pretransplant hematological parameters

**DOI:** 10.1097/MD.0000000000048804

**Published:** 2026-05-29

**Authors:** Min Zhu, Feng Wang

**Affiliations:** a Department of Nephrology, Shulan (Hangzhou) Hospital, Hangzhou City, Zhejiang Province, P.R. China.

**Keywords:** delayed graft function, hematological parameters, kidney transplantation, renal artery resistive index, risk prediction model

## Abstract

Delayed graft function (DGF) remains a frequent early complication after kidney transplantation and is associated with prolonged hospitalization and inferior graft outcomes. This retrospective cohort study evaluated whether the renal artery resistive index (RI) combined with pretransplant hematological parameters could improve DGF prediction. Adult recipients who underwent kidney transplantation between January 2022 and December 2024 at Shulan (Hangzhou) Hospital were screened; 280 patients were included and categorized into a DGF group (n = 88) or a non-DGF group (n = 192) according to the requirement for dialysis within the first posttransplant week. Pretransplant blood tests and postoperative Doppler-derived RI were collected. Univariate analyses demonstrated significant between-group differences in RI, serum creatinine, blood urea nitrogen, and albumin, whereas demographic factors, primary kidney disease, dialysis characteristics, leukocyte indices, and immunosuppressive regimen did not differ significantly. In multivariable logistic regression, RI, serum creatinine, blood urea nitrogen, and albumin independently predicted DGF. Receiver operating characteristic analysis showed strong discriminatory performance for the combined model (area under the curve = 0.923; 95% confidence interval: 0.888–0.957), yielding high sensitivity (90.91%) and moderate specificity (79.69%). Stratification by the optimal combined-indicator cutoff further demonstrated associations with postoperative organ dysfunction severity and patient-reported quality of life. These findings support incorporating RI with routine hematological parameters to facilitate early risk stratification for DGF after kidney transplantation.

## 1. Introduction

In 2022, the field of kidney transplantation has made significant advancements amidst a complex and evolving medical landscape, achieving remarkable milestones while also facing numerous challenges. In the United States, the total number of kidney transplants reached a record high of 26,309 cases,^[1]^ mainly attributed to the continuous growth of deceased donor kidney transplants, reflecting the resilience of organ transplantation efforts in the face of adverse factors like the pandemic. However, the development in this field is not without hurdles, as there are still many pressing issues to be addressed.^[2]^ Delayed graft function (DGF) is a common early complication after kidney transplantation and is associated with prolonged hospitalization and poor graft outcomes. Early identification of patients at high risk of DGF is essential for optimizing perioperative management and improving prognosis.^[3]^ Current prediction approaches are limited and often rely on single indicators, which may not fully capture the complex pathophysiology of DGF. The renal artery resistive index (RI), assessed by Doppler ultrasound, reflects graft hemodynamics, while hematological parameters such as serum creatinine (Scr), blood urea nitrogen (BUN), and albumin (ALB) reflect renal function and systemic condition.^[4–6]^ However, evidence on the combined predictive value of these indicators remains limited.^[7,8]^ Therefore, this study aimed to develop a predictive model integrating RI and pretransplant hematological parameters to improve early risk stratification for DGF after kidney transplantation.

## 2. Methods

### 2.1. Study design and population

**Figure 1.**
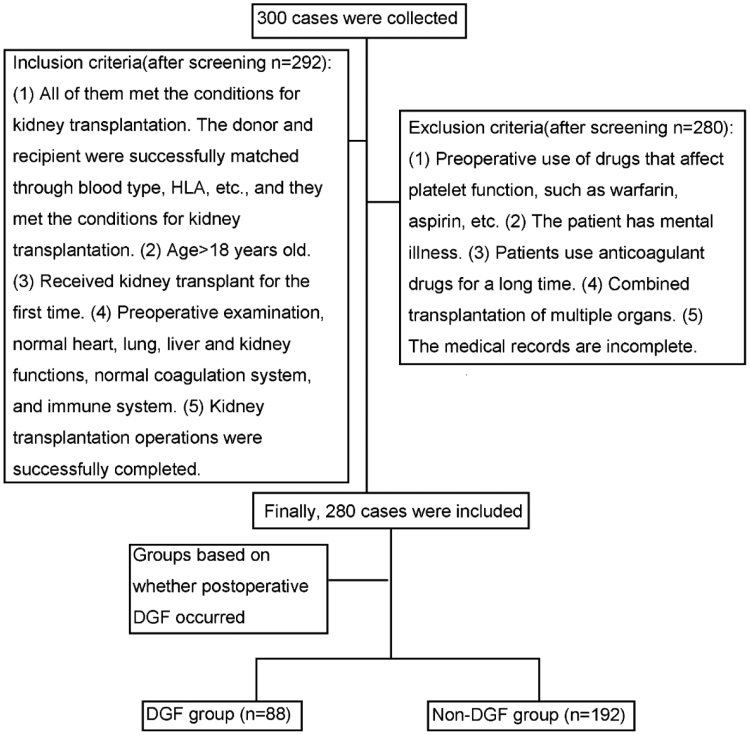
Flow diagram of the patients included. DGF = delayed graft function, HLA = human leukocyte antigen.

This study was approved by the Ethics Committee of Shulan (Hangzhou) Hospital. Sample size calculation was performed using the formula N = Z_α/2_^2^π (1 − π)/δ^2^, where based on previous literature, the occurrence rate of DGF after kidney transplantation was estimated at π = 31%.^[7]^ With a significance level of α = 0.05 and an allowable error of δ = 0.06, the calculated minimum sample size was approximately 229. Considering the possibility of invalid samples, the sample size was expanded by 20%, resulting in a minimum of 275 samples. A total of 300 patients who underwent kidney transplantation in our hospital from January 2022 to December 2024 were retrospectively selected. After screening, 280 cases were finally included. Patients were divided into 2 groups based on whether DGF occurred postoperatively: the DGF group (n = 88) and the non-DGF group (n = 192). The criteria for defining DGF included the need for dialysis within the first week after kidney transplantation.^[8]^ The inclusion criteria were as follows: meeting the conditions for kidney transplantation, successful matching of donor and recipient through blood type, human leukocyte antigen, and other examinations, and meeting the requirements for kidney transplantation; age > 18 years; first-time kidney transplantation recipients; normal preoperative examinations with normal cardiac, pulmonary, hepatic, renal, coagulation, and immune system functions; and successful completion of the kidney transplantation procedure. The exclusion criteria were as follows: preoperative use of platelet-affecting drugs such as warfarin and aspirin, presence of psychiatric disorders, long-term use of anticoagulants, multiple organ transplants, and incomplete medical records (Fig. 1). This study was approved by the Ethics Committee of Shulan (Hangzhou) Hospital. The procedures were conducted in accordance with the ethical standards set forth by the Committee on Human Experimentation and the Helsinki Declaration of 1964, as revised in 2013. All patients have signed informed consent forms. This study was designed as a retrospective model development study. Due to the single-center nature and limited sample size, no independent validation cohort was included. Future multicenter studies with external validation cohorts are required to confirm the robustness and generalizability of the model. Cases with incomplete clinical or laboratory data were excluded from analysis. No imputation methods were applied.

### 2.2. Data collection

Patient general information was collected with the electronic medical record system. Before transplantation, venous blood samples were collected from all patients for the measurement of white blood cell count, neutrophils, lymphocytes, monocytes, Scr, BUN, and ALB. Cases with incomplete clinical or laboratory data were excluded from analysis, and no imputation methods were applied. To reduce measurement bias, ultrasound assessments were performed by experienced operators following standardized protocols.

### 2.3. Ultrasound examination

Ultrasound examinations were conducted using the Philips iU22 or HDI 5000 color Doppler ultrasound diagnostic equipment (Philips Healthcare, Bothell) equipped with a low-frequency convex array probe, with the operating frequency set between 3 and 5 MHz. Patients were required to fast before the examination and assume a supine or left lateral position for observation. Ultrasound was performed to assess the renal graft, including measurement of RI using standard Doppler techniques. To ensure consistency in ultrasound examinations, all experimental ultrasound data were obtained by experienced senior staff at our hospital. Personnel underwent regular training assessments to enhance their professional skills, standardized operational guidelines were established to regulate their techniques, and a supervisory mechanism was implemented to ensure quality control throughout the examination process, thereby guaranteeing the accuracy and consistency of each ultrasound examination.

### 2.4. Prognostic indicators

Sequential Organ Failure Assessment (SOFA)^[9]^: the SOFA score uses 6 criteria to reflect the functionality of organ systems (respiratory, hematologic, liver, cardiovascular, neurological, and renal systems), with each criterion scored from 0 to 4. Higher scores indicate a poorer prognosis. Evaluation was conducted using the European Organization for Research and Treatment of Cancer Quality of Life Questionnaire-Core 30.^[10]^ It consists of 9 items, each scored from 1 to 10, with higher scores indicating a better quality of life.

### 2.5. Statistical analysis

The experimental data collected were analyzed with Statistical Package for the Social Sciences 27.0 (International Business Machines Corporation, Armonk). The Shapiro–Wilk test was used for normality testing. For normally distributed continuous data, results were presented as mean ± standard deviation, and comparisons were made with an independent-samples *t* test. Count data were presented as frequencies or rates, and comparisons were conducted with the χ^2^ test or the Fisher exact test. Influencing factors were analyzed using univariate and binary logistic regression analysis. The predictive value of relevant indicators for the occurrence of DGF after kidney transplantation in patients was evaluated using the receiver operating characteristic (ROC) curve. A significance level of *P* < .05 was considered statistically significant for differences. In the multivariable model, 4 predictors were included with 88 events, resulting in an events-per-variable ratio of 22, which meets the recommended threshold for stable model estimation.

## 3. Results

### 3.1. Univariate analysis of factors influencing occurrence of DGF in kidney transplantation patients

Comparison of age, gender, body mass index, primary disease, smoking history, alcohol history, preoperative immunosuppressive regimen, dialysis time, dialysis modality, white blood cell count, neutrophils, lymphocytes, and monocytes between the 2 groups showed no statistically significant differences (*P* > .05). However, comparisons of RI, Scr, BUN, and ALB revealed statistically significant differences (*P* < .05; Table 1).

**Table 1:** Univariate analysis of factors influencing occurrence of DGF in kidney transplantation patients.

Indicator	DGF group (n = 88)	Non-DGF group (n = 192)	*t/*χ^2^ value	*P* value
Age (yr)	46.68 ± 3.54	47.02 ± 4.21	0.658	.511
Gender				
Male	60	124	0.347	.556
Female	28	68		
BMI (kg/m^2^)	21.54 ± 1.31	21.65 ± 1.73	0.531	.596
Primary disease				
Glomerulonephritis	53	110	1.134	.889
Diabetic nephropathy	11	20		
Hydronephrosis	6	13		
Hypertensive nephropathy	8	19		
Others	10	30		
Smoking history				
Yes	31	65	0.050	.822
No	57	127		
Alcohol-drinking history				
Yes	36	70	0.508	.476
No	52	122		
Preoperative immunosuppressive regimen				
Belatacept	67	142	0.151	.697
Antithymocyte globulin	21	50		
Dialysis time (mo)	48.54 ± 3.45	49.09 ± 3.96	1.122	.263
Dialysis modality				
Hemodialysis	79	170	0.093	.761
Peritoneal dialysis	9	22		
RI	0.70 ± 0.08	0.60 ± 0.07	10.601	<.001
WBC (×10^9^/L)	7.16 ± 1.13	6.97 ± 1.06	1.364	.174
NEU (×10^9^/L)	5.34 ± 1.01	5.13 ± 1.09	1.531	.127
LYM (×10^9^/L)	1.35 ± 0.24	1.34 ± 0.37	0.232	.817
MONO (×10^9^/L)	0.46 ± 0.14	0.43 ± 0.12	1.841	.067
Scr (μmol/L)	621.22 ± 157.24	531.03 ± 159.53	4.411	<.001
BUN (μmol/L)	394.77 ± 62.20	341.47 ± 53.69	7.329	<.001
ALB (g/L)	35.48 ± 2.32	37.41 ± 2.15	6.800	<.001

ALB = albumin, BMI = body mass index, BUN = blood urea nitrogen, DGF = delayed graft function, LYM = lymphocytes, MONO = monocytes, NEU = neutrophils, RI = renal artery resistive index, Scr = serum creatinine, WBC = white blood cell count.

### 3.2. Binary logistics regression analysis of factors influencing occurrence of DGF in kidney transplantation patients

After conducting collinearity analysis on the variables that were significant in the univariate analysis, these variables were set as independent variables, and their original values were included. The occurrence of DGF postoperatively was set as the dependent variable (DGF = 1, non-DGF = 0) for analysis. The results of the binary logistics regression analysis indicated that RI, Scr, BUN, and ALB were independent influencing factors for the occurrence of DGF in kidney transplantation patients (*P* < .05; Table 2).

**Table 2:** Binary logistics regression analysis of factors influencing occurrence of DGF in kidney transplantation patients.

Factor	β	Standard error	Wald	*P*	Exp(β)	95% CI		Collinearity analysis	
						Lower limit	Upper limit	Tolerance	VIF
RI	0.220	0.033	43.922	<.001	1.246	1.167	1.330	0.949	1.053
Scr	0.005	0.001	12.564	<.001	1.005	1.002	1.007	0.959	1.043
BUN	0.015	0.003	20.186	<.001	1.015	1.009	1.022	0.912	1.097
ALB	−0.399	0.088	20.394	<.001	0.671	0.565	0.798	0.952	1.050
Constant	−8.724	3.845	5.149	.023	<0.001	–	–	–	–

ALB = albumin, BUN = blood urea nitrogen, CI = confidence interval, DGF = delayed graft function, RI = renal artery resistive index, Scr = serum creatinine, VIF = variance inflation factor.

### 3.3. ROC curve analysis of predictive value of indicators

**Figure 2.**
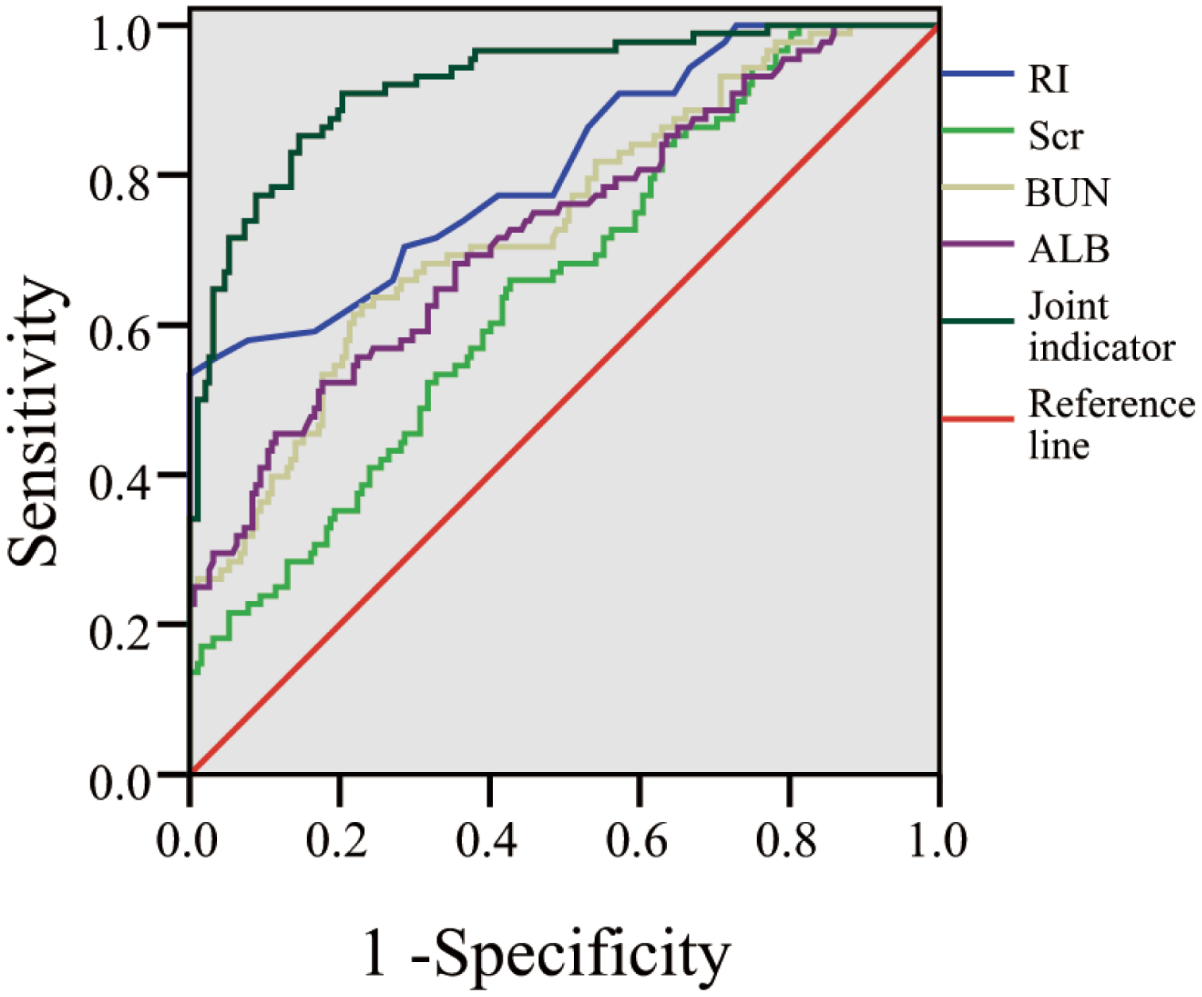
ROC curve analysis of predictive value of indicators. ALB = albumin, BUN = blood urea nitrogen, RI = renal artery resistive index, ROC = receiver operating characteristic, Scr = serum creatinine.

The ROC analysis results indicated that the area under the curve (AUC) for the combined indicators was 0.923, with a standard error of 0.017 (95% confidence interval [CI]: 0.888–0.957, *P* < .001). The Youden index was 0.71, with a sensitivity of 90.91% and specificity of 79.69%. Among the single indicators, RI exhibited the strongest diagnostic ability with an AUC of 0.811 (95% CI: 0.755–0.868) and a Youden index of 0.52. At its optimal cutoff (0.71), it yielded very high specificity (96.34%), albeit with lower sensitivity (55.68%). BUN ranked next with an AUC of 0.736 (95% CI: 0.673–0.800) and a Youden index of 0.40. At the optimal cutoff value of 385.02, sensitivity was 62.50%, and specificity was 77.08%. ALB had an AUC of 0.725 (95% CI: 0.660–0.791) and a Youden index of 0.35. At its optimal cutoff (35.71), sensitivity was 52.27%, and specificity was 82.29%. Scr showed the relatively lowest diagnostic performance among the single indicators, with an AUC of 0.653 (95% CI: 0.586–0.720) and a Youden index of 0.23. At its optimal cutoff (559.21), sensitivity was 65.91%, and specificity was 57.29%. The AUC for all assessed indicators was statistically highly significant (*P* < .001), indicating diagnostic utility (Fig. 2).

### 3.4. Comparison of SOFA scores and quality of life scores in patients with different optimal cutoff values of combined indicators

Patients in the group with combined indicators ≤0.25 had lower SOFA scores and higher European Organization for Research and Treatment of Cancer Quality of Life Questionnaire-Core 30 (EORTC QLQ-C30) scores compared with those in the group with combined indicators >0.25, showing statistically significant differences (*P* < .05; Table 3).

**Table 3:** Comparison of SOFA scores and quality of life scores in patients with different optimal cutoff values of combined indicators.

	Count	SOFA		EORTC QLQ-C30	
		Pretreatment	Posttreatment	Pretreatment	Posttreatment
Combined indicators ≤0.25	159	15.64 ± 1.35	17.65 ± 1.17	55.65 ± 10.54	71.54 ± 10.61
Combined indicators >0.25	121	15.51 ± 1.54	16.54 ± 1.34	56.07 ± 10.33	64.34 ± 10.34
*t* value		0.751	7.383	0.333	5.687
*P* value		.453	<.001	.739	<.001

EORTC QLQ-C30 = European Organization for Research and Treatment of Cancer Quality of Life Questionnaire-Core 30, SOFA = Sequential Organ Failure Assessment.

## 4. Discussion

This study focused on the predictive value of ultrasound-assessed RI combined with hematological indicators for the occurrence of DGF after kidney transplantation, aiming to provide clinical references for early, precise prediction and timely intervention to improve the prognosis of kidney transplantation recipients. In the univariate analysis, significant differences were found in the comparison of RI, Scr, BUN, and ALB between the 2 groups of patients, indicating a potential association of these indicators with the occurrence of DGF. Further binary logistic regression analysis confirmed that RI, Scr, BUN, and ALB were independent influencing factors for the occurrence of DGF in kidney transplantation patients. RI is a crucial ultrasound indicator reflecting transplant kidney perfusion, and higher values may signify increased vascular resistance and inadequate blood perfusion, thereby affecting kidney function recovery. The results of this study are consistent with the study by Guo et al,^[11]^ further confirming the significant role of RI in predicting DGF. Analyzing the potential mechanisms, from a hemodynamic perspective, DGF fundamentally represents an acute kidney injury state where pathological mechanisms such as donor inflammatory status, ischemia/reperfusion injury, and activation of immune responses can impair the microcirculation of the transplanted kidney.^[12,13]^ After kidney transplantation, changes occur in renal blood perfusion, leading to alterations in renal vascular resistance. RI reflects the resistance of renal blood vessels, and when the renal microcirculation is impaired, vascular resistance increases, resulting in elevated RI values. Studies by Mwipatayi et al^[14]^ have shown a significant correlation between RI elevation within 24 hours posttransplantation and DGF, indicating that this may be due to the early response to stress such as surgical trauma and ischemia-reperfusion in the kidneys, during which renal vascular resistance undergoes rapid alterations. Due to its ability to promptly capture these hemodynamic changes, RI may serve as an effective early marker for identifying high-risk allografts. Scr and BUN are commonly used clinical indicators for assessing kidney function, with elevated levels typically indicating impaired kidney function. Previous studies, such as the one by Parajul et al,^[15]^ have demonstrated the value of Scr in predicting DGF after kidney transplantation. However, the research by Parajul et al suggested that the application value of serum β2-microglobulin in DGF surpasses that of Scr, although its mechanism remains unclear and warrants further investigation. The dynamic changes in Scr and BUN can reflect the functional status of the transplanted kidney and are closely related to the occurrence of DGF. A study by Li et al^[16]^ also indicated the predictive value of Scr and BUN in DGF after kidney transplantation, aligning with the viewpoint of this study, further substantiating the predictive value of Scr and BUN. Moreover, a decrease in ALB levels may reflect poor nutritional status or the presence of inflammation in patients. These factors could potentially affect the repair and functional recovery of the transplanted kidney, thereby increasing the risk of DGF occurrence.

The combined ROC analysis of RI, Scr, BUN, and ALB yielded an AUC of 0.923, with a standard error of 0.017 (95% CI: 0.888–0.957). The Youden index was 0.71, with a sensitivity of 90.91% and specificity of 79.69%. These results indicate that the combined indicators have high predictive value for the occurrence of DGF in kidney transplant patients. Compared with single indicators, the combined indicators can comprehensively consider multiple aspects of information, providing a more comprehensive reflection of the patient’s condition and the functional status of the transplanted kidney, thereby enhancing prediction accuracy. In clinical practices, accurate prediction of DGF occurrence is crucial for timely intervention and improving patient outcomes.^[17,18]^ By monitoring the combined indicators, physicians can identify high-risk patients for DGF early postoperatively and devise personalized treatment plans such as adjusting immunosuppressive regimens and enhancing nutritional support to reduce the incidence and severity of DGF.

Furthermore, this study observed that patients in the group with combined indicators ≤0.25 had lower SOFA scores compared with those in the >0.25 group, while their EORTC QLQ-C30 scores were higher. These differences were statistically significant. The SOFA score is used to assess the severity of organ dysfunction, where lower scores indicate milder dysfunction. On the contrary, the EORTC QLQ-C30 is a scale for evaluating patients’ quality of life, where higher scores reflect better quality of life. These findings suggest that the combined indicators are not only associated with the occurrence of DGF but may also impact the severity of the patient’s condition and quality of life. Patients with lower combined indicator scores exhibited relatively milder conditions and higher quality of life, underscoring the importance of early prediction and intervention for DGF. Early detection and timely intervention can aid in improving patient outcomes.^[19,20]^ However, this study has certain limitations. First, being a retrospective study, there may be such issues as selection bias and information bias. Second, although the sample size is relatively large, there may still be limitations, and future larger-scale, multicenter prospective studies are needed to further validate the conclusions of this study. In addition, this study only explored the relationship between some ultrasound and hematological indicators and DGF, and there may be other factors that were not included in the study. Further in-depth research is required to comprehensively understand the mechanisms and influencing factors of DGF.

## 5. Conclusion

In conclusion, the combination of RI with hematological indicators holds significant application value in predicting DGF after kidney transplantation. It is recommended to include RI, Scr, BUN, and ALB in the indicators for predicting DGF after kidney transplantation. In clinical practices, attention should be paid to the monitoring and analysis of these indicators to early identify high-risk patients for DGF and implement effective intervention measures, thereby enhancing the success rate of kidney transplantation and the quality of patient survival. Furthermore, future research efforts should focus on further refining predictive models and treatment strategies for DGF.

## Acknowledgments

The authors are grateful to all participants in the present study.

## Author contributions

**Conceptualization:** Min Zhu, Feng Wang.

**Data curation:** Min Zhu, Feng Wang.

**Formal analysis:** Min Zhu, Feng Wang.

**Funding acquisition:** Feng Wang.

**Investigation:** Feng Wang.

**Writing – original draft:** Feng Wang.

**Writing – review & editing:** Feng Wang.
